# Case Report: Plasmacytoma of External Urethral Meatus

**DOI:** 10.3389/fendo.2022.783855

**Published:** 2022-02-16

**Authors:** Wen Su, Liang Zeng, Dongsheng Zhao, Ying Fu, Jin Tang

**Affiliations:** ^1^ Department of Urology, The Third Xiangya Hospital, Central South University, Changsha, China; ^2^ Department of Nuclear Medicine, Hunan Cancer Hospital Affiliated to Central South University, Changsha, China

**Keywords:** plasmacytoma, plasma cells, urethra, radiotherapy, surgery

## Abstract

**Rationale:**

Extramedullary plasmacytoma (EMP) can occur in various parts of the body. It is generally accepted that the highest site of occurrence is the head and neck region (80% to 90%), followed by the gastrointestinal tract and the skin. It is worth mentioning that the site of disease, in this case, was the urethral meatus, which is extremely rare in clinical practice.

**Patient Concerns:**

A 50-year-old female complained of an episode of painless gross hematuria without symptoms of frequent urination, urgency, abdominal pain, abdominal distension, fever, or oliguria. The patient has no history of smoking or drinking and denied any family history of solid malignancy or hematological disease.

**Diagnosis and Interventions:**

Urethrocystoscopy revealed urethral polypoid hyperplasia, which we initially thought could be a urethral caruncle. The patient was asked to undergo caruncle resection after 1 week of potassium permanganate sitz bath, and postoperative pathology revealed plasmacytoma. After that, a whole-body MRI showed no other lesions. She received postoperative radiotherapy.

**Outcomes:**

During radiotherapy, the patient’s condition and appetite were fair, and the skin mucositis was I°. Pelvic contrast-enhanced MRI and whole-body PET-CT 6 months after urethral meatus lumpectomy and radiotherapy showed changes without obvious abnormal signs. No recurrence or metastasis was detected after one year of follow-up.

**Lessons:**

Urinary EMP is a rare disease. It is not easy to diagnose due to its rare site of occurrence and non-specific symptoms. The diagnosis of EMP requires a combination of imaging studies and pathological findings. Currently, surgery combined with radiotherapy or radiotherapy alone is the mainstay treatment and usually results in an acceptable local control rate. At the same time, chemotherapy cannot be ignored.

## Introduction

Plasmacytomas include solitary plasmacytomas, extramedullary plasmacytomas (EMP), multiple myeloma (MM), and plasma cell leukemia. EMP is a rare malignant tumor arising in the extramedullary soft tissue. It accounts for approximately 4% of all plasmacytomas, including 80% in the upper respiratory and digestive tracts and a very rare occurrence in the urinary tract (1). We report the first case of plasmacytoma of the urethral meatus, and the rest of the urethra in this patient was unremarkable. EMP is defined as a plasma cell tumor arising outside the hematopoietic tissue of the bone marrow, and it is a rare form of malignant monoclonal plasma cell lesions. The age of onset of EMP ranges from 55 to 60 years. The vast majority (75% to 86%) occur in men, and the incidence increases with age (2). Based on the literature search, studies on only 9 similar cases with such neoplasms have been reported to date (3–5). Notably, the causative site, in this case, was the external urethral meatus, which is rare in clinical practice. The patient in this report was treated by surgery and radiotherapy.

## Case Description

**Figure 1:**
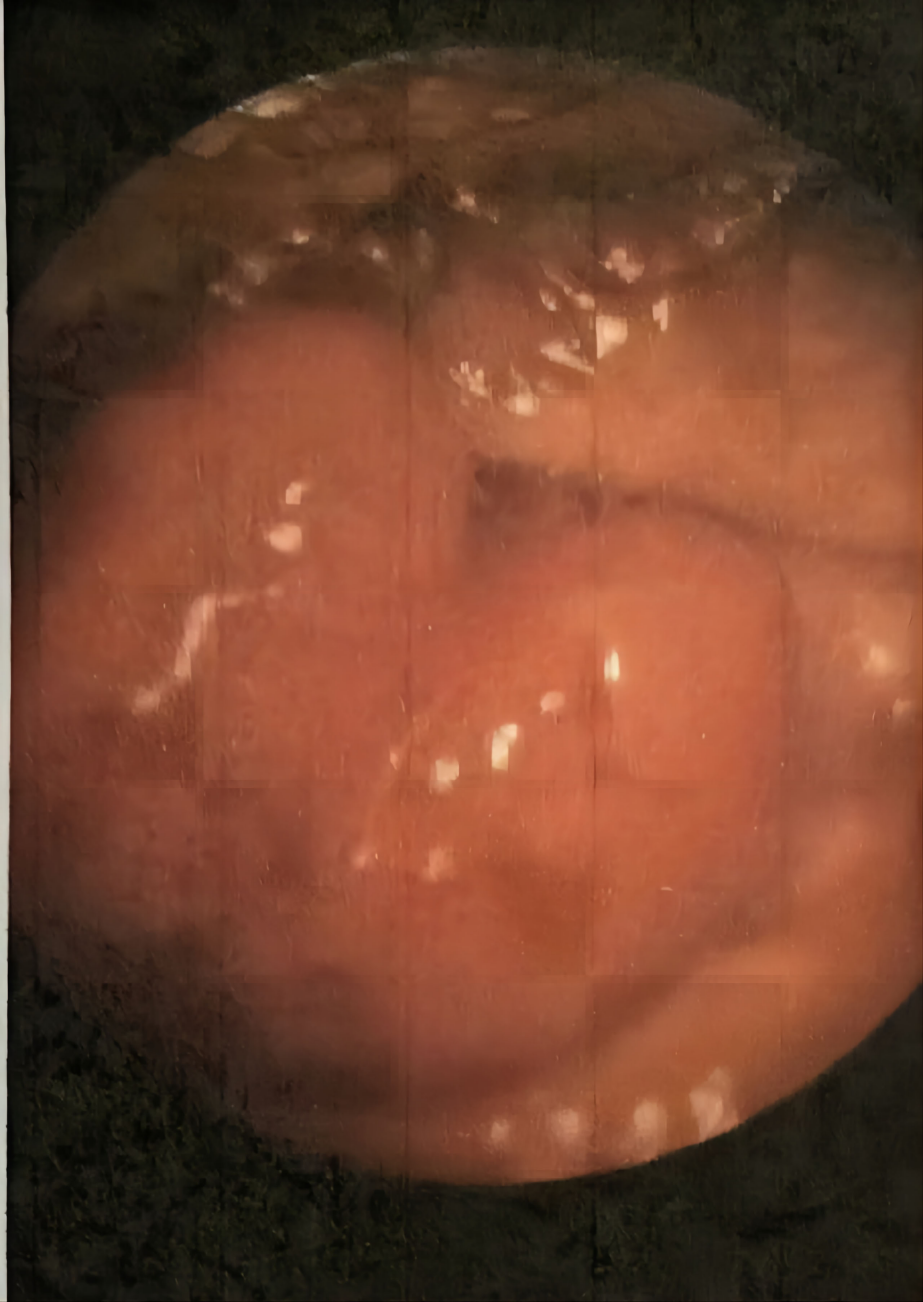
A polypoid hyperplasia of 2 cm ✖ 1.5 cm in size was observed at 6 o'clock in the external urethral meatus, with smooth surface.

A 50-year-old female presented with a chief complaint of an episode of painless gross hematuria. On specialist physical examination, a bulge about 2 *2 cm in size was macroscopically observed in the external urethral meatus, without skin ulceration, tenderness, soft, and the rest were unremarkable. She had no specific past medical history. Thus, she underwent urethrocystoscopy in the outpatient clinic, which revealed a urethral meatus neoplasm (**Figure 1**). A polypoid hyperplasia of 2 cm × 1.5 cm in size was observed at 6 o’clock in the external urethral meatus with a smooth surface. The patient was asked to perform a potassium permanganate sitz bath by herself at home once a day for 1 week, and she was then admitted for further treatment. After admission, preoperative examinations (chest radiography and electrocardiogram) were carried out without significant abnormalities, and tumor resection was performed under spinal anesthesia. During the operation, the tumor was seen to have a clear boundary with the surrounding. It was completely removed to the normal urethra mucosa level without significant abnormalities in the urethra.

**Figure 2:**
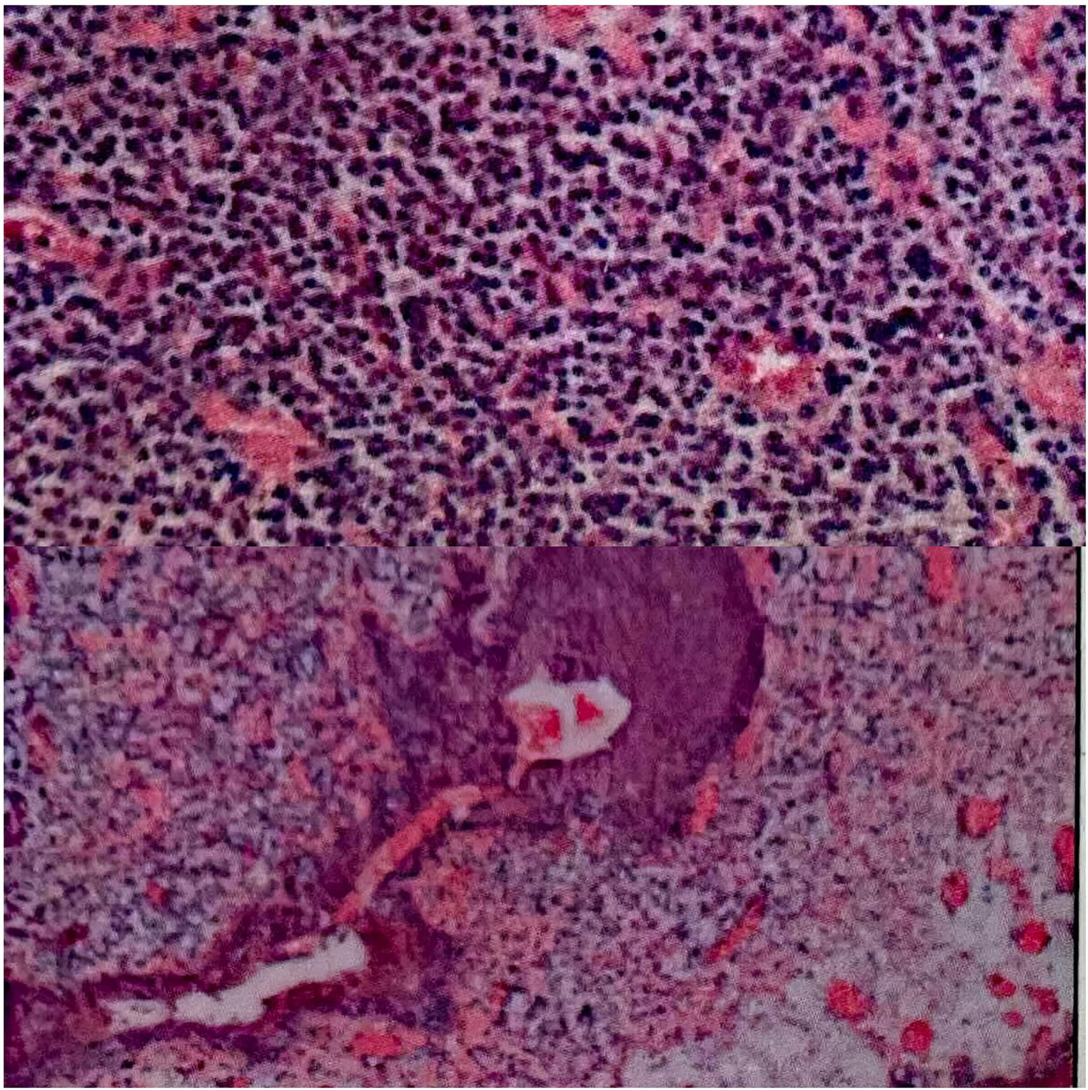
(Urethral mass) submitted tissue showed polypoid growth, extensive scaling of the surface epithelium, invagination of the regional transitional epithelium, glandular arrangement, and patchy hyperplasia of plasmacytoid cells in the lamina propria.

Postoperative pathological diagnosis (**Figure 2**): The plasma cells in the lamina propria of the submitted tissue proliferated in sheets, and well-differentiated plasmacytoma was considered in combination with immunohistochemistry. Immunohistochemistry: tumor cells CD38 (+), CD138 (+), MUM1 (+), Kappa (+), CD79α (+), CD19 (+), Bcl-2 (+), Lambda (-), CD3 (-), CD20 (-), CD5 (-), Cyclin D1 (-), CKpan (-), CD10 (-), CD21 (-), CD56 (-) TdT (-), Ki67 (about 10%).

According to the above results, the patient was preliminarily diagnosed with a plasma cell neoplasm. So we continued to improve the relevant examinations such as blood and bone marrow except for the cumulative possibility of multiple myeloma. β-2 microglobulin decreased, and serum alkaline phosphatase, free monoclonal light chain assay, blood, urine protein electrophoresis, and bone marrow biopsy were negative, making the diagnosis a solitary EMP. At the same time, we also did a whole-body MRI, which showed no obvious lesions. We then contacted the oncologist to develop the next radiotherapy plan. At present, the patient is followed up for one year after radiotherapy, and there is no recurrent lesion in the whole body.

## Discussion

EMP is an extraosseous or extramedullary tumor formed by monoclonal proliferation of B lymphocytes, generally without myeloma-related clinical manifestations and related laboratory positive indicators such as anemia, excessive monoclonal immunoglobulin in serum or urine, hypercalcemia, and renal failure. The clinical manifestations of EMP vary according to the site of tumor onset, lack of specificity, and most present with pain and discomfort at the lesion site due to local tumor compression. Some patients have palpable tumors during physical examination. The tumor in this patient was located in the urethral meatus, which is unprecedented in clinical practice. This tumor location increases the difficulty of diagnosis. The patient in this case had a preoperative diagnosis of female urethral caruncle without associated imaging studies. Postoperative pathological examination revealed plasmacytoma.

It is generally believed that EMP may be occult MM. The diagnosis of EMPs is problematic because it requires confirmation by extensive radiology, hematology, and histopathology (6–8). At present, the diagnostic criteria for EMP have not been unified, but the principal bases are I primary extramedullary plasmacytoma confirmed by pathology; II bone marrow plasma cell infiltration occupying less than 5% of nuclear cells; III normal skeletal imaging examination with no lytic changes; IV anemia, hypercalcemia and renal insufficiency caused by no plasmacytosis; V no M protein in serum or a small amount of M protein detected (9). Systemic bone involvement of the bone marrow must be excluded and, therefore, a bone marrow biopsy is also necessary. The patient in this study underwent a pathological resection of the biopsy, differential examinations such as whole-body DWI and bone marrow biopsy, which ruled out MM and confirmed the clinical diagnosis.

Primary urethral plasmacytoma is a rare tumor, and to our knowledge, this patient is the first case of plasmacytoma to occur in the external urethral meatus, and the patient had no abnormalities in the urethra. No definitive treatment strategy exists. There are currently three main treatment modalities for localized EMP, radiotherapy, surgical treatment, and postoperative radiotherapy. As for chemotherapy, it is generally used for patients with recurrence after treatment or systemic metastasis (10). The chemotherapy regimen can refer to the chemotherapy regimen for multiple myeloma. Radiotherapy is the main treatment for EMP, especially for surgical resection of EMP with large size or close to vital organs. Radiotherapy is a very important choice, which can achieve good local control and disease-free survival rate. In selecting radiotherapy dose, it is generally believed that 40 ~ 50 Gy can achieve a better local control rate without causing serious radiation damage (11–14). Mendenhall et al. (15) found that local control rates of 94% were reported at radiation doses higher than 40 Gy but 69% at doses lower than 40 Gy. Our patient had total radiotherapy up to: PTV D95% = 43.2 Gy/24 fractions; PTGV D95% = 48 Gy/24 fractions. We used 60MV-X-ray for three-dimensional intensity-modulated radiotherapy, in which GTVtb was the extent of urethral meatus disease, PGTV was GTVtb placed 5 mm externally, and CTV included PGTV placed 1 cm externally including the posterior urethra of the bladder triangle.

Some studies have found that lymph node metastasis and primary tumors larger than 5 cm are independent poor prognostic factors for EMP (16). The prognosis of EMP is that the 5-year survival rate can reach 90%, the 10-year disease-free survival rate can reach 70%, and the 10-year progression to multiple myeloma is 11% to 30%. The Surveillance, Epidemiology, and End Results database included 1185 patients with EMP and reported better survival in patients with head and neck involvement than those with other sites (13 vs. 4 years). EMP patients included in this database were more likely to receive combination therapy (surgery and RT) than surgery alone (17, 18). Therefore, no clear treatment strategy exists. The histological appearance of the tumor at the time of surgery was not suggestive of aggressive behavior. However, as transmission may occur years later and the final outcome is unpredictable, we will continue to track it.

In conclusion, EMP in women is extremely rare, especially in the urethral meatus. EMP can either recur locally or progress to MM. Based on this case, it seems slightly inappropriate to operate without perfecting the imaging examination; therefore, we concluded that in the case of the first diagnosis of urethral lesions, the imaging examination of at least the pelvic part should be done as far as possible. Fortunately, thanks to timely postoperative diagnosis and treatment, the patient’s current disease control is fair. Also, we would like to emphasize the importance of multidisciplinary collaboration in the treatment process.

## Data Availability Statement

The original contributions presented in the study are included in the article/supplementary material. Further inquiries can be directed to the corresponding author.

## Ethics Statement

Written informed consent was obtained from the patient for the publication of any potentially identifiable images or data included in this article.

## Author Contributions

JT: Revising the manuscript critically for important intellectual content. WS: Drafting the manuscript. LZ and DZ: Data collection and sorting. YF: Provide valuable opinions in the interdisciplinary field. All authors contributed to the article and approved the submitted version.

## Conflict of Interest

The authors declare that the research was conducted in the absence of any commercial or financial relationships that could be construed as a potential conflict of interest.

## Publisher’s Note

All claims expressed in this article are solely those of the authors and do not necessarily represent those of their affiliated organizations, or those of the publisher, the editors and the reviewers. Any product that may be evaluated in this article, or claim that may be made by its manufacturer, is not guaranteed or endorsed by the publisher.
